# Posterior Fixation Combined with Anterior Transoral Plate Osteosynthesis for Chronic Gehweiler IIIb Atlas Fracture–Dislocation: Case Report and Literature Review

**DOI:** 10.3390/jcm15145522

**Published:** 2026-07-14

**Authors:** Qingfeng Shen, Xiaoming Tian, Shibo Ma, Wenbin Xue, Hua Wei, Junwei Gao, Haifeng Song, Yingpeng Xia

**Affiliations:** 1 Department of Spine Surgery, Tianjin Union Medical Center, The First Affiliated Hospital of Nankai University, Nankai University, 190 Jieyuan Road, Tianjin 300121, China; dshenqingfeng@sina.com (Q.S.); xiaoming-tian@hotmail.com (X.T.); mashibo2010@hotmail.com (S.M.); gaojunwei41@163.com (J.G.); drsonghf@163.com (H.S.); 2 Tianjin Institute of Spinal Surgery, Tianjin 300121, China; 3 School of Medicine, Nankai University, 94 Weijin Road, Tianjin 300071, China; xuewenbin0705@163.com; 4 The First Hospital of Hebei Medical University, Hebei Medical University, 89 Donggang Road, Shijiazhuang 050023, China; wh908120@163.com

**Keywords:** atlas fracture, internal fixation, transoral approach, osteosynthesis plate, case report

## Abstract

**Objective:** This study aims to report a clinical case involving posterior fixation combined with anterior transoral plate osteosynthesis in the management of chronic Gehweiler IIIb atlas fracture–dislocation, thereby providing a reference for similar cases. **Methods:** A patient with atlas fracture–dislocation following a fall injury underwent surgical intervention. Preoperative diagnosis confirmed Gehweiler IIIb fracture with right atlantoaxial lateral mass dislocation. The treatment protocol involved posterior reduction, instrumentation, and anterior transoral screw–plate fixation. **Results:** The postoperative recovery was uneventful, with significant alleviation of cervical pain. The most recent follow-up assessment revealed favorable osseous union and appropriate stabilization of the internal implants. The VAS score decreased from 4 preoperatively to 0 postoperatively, and the NDI decreased from 64% to 16%, concurrent with a notable enhancement in the patient’s quality of life. **Conclusions:** Combined posterior fixation and anterior transoral plate osteosynthesis demonstrates efficacy in ameliorating clinical manifestations and facilitating osseous consolidation in cases of chronic Gehweiler IIIb atlas fracture–dislocation. This surgical strategy demonstrates favorable therapeutic outcomes for refractory atlas fractures and warrants clinical application in analogous scenarios, but still requires extensive clinical verification.

## 1. Introduction

Upper cervical spine injuries resulting from falls, diving accidents, or vehicular collisions typically occur due to external forces transmitted to the craniocervical complex [1]. The biomechanical principle underlying atlas burst fractures was initially described by Jefferson: when vertical compressive loading (axial force) is applied to the atlas, the force transmission pathway becomes altered, resulting in outward displacement of bilateral lateral masses [2].

The classification principles for atlantoaxial fractures are diverse [3], and the management of atlas fractures primarily involves conservative treatment (external fixation) rather than surgical intervention (internal fixation), though therapeutic strategies remain controversial [4]. For stable atlas fractures, conservative management is widely regarded as the preferable option [5]. However, unstable fractures often demonstrate poor clinical outcomes with external fixation alone. Traditional surgical approaches predominantly rely on posterior atlantoaxial fusion or occipitocervical fixation, which achieve satisfactory stability but restrict cervical mobility. Given the pivotal role of the atlantoaxial complex in cervical rotation, posterior instrumentation frequently leads to reductions in postoperative quality of life. Anterior transoral approaches have gained increasing attention in recent decades. The earliest documented transoral intervention dates to 1917, when Kanavel successfully extracted a bullet fragment from the anterior arch of the atlas via this route [6]. While the transoral approach for upper cervical spine access was first proposed in 1935 [7], the technique for anterior transoral internal fixation was not established until 2004 by Michael Ruf [8]. Although the transoral approach provides direct access to the anterior spinal column of the upper cervical vertebrae through straightforward exposure and minimal tissue dissection, its clinical implementation remains limited due to the low prevalence of upper cervical pathologies requiring anterior intervention; the anatomical complexity of the craniocervical junction; challenges posed by the oropharyngeal microenvironment; and technical demands in postoperative management (infection control, airway maintenance). These factors collectively hinder its widespread adoption in routine practice.

Despite considerable research on surgical approaches for atlas fractures, no consensus exists regarding the optimal management of irreducible chronic atlas fractures with lateral mass dislocation. This study presents a therapeutic strategy employing posterior fixation combined with anterior transoral plate osteosynthesis in a patient with chronic Gehweiler IIIb atlas fracture–dislocation with fibrotic non-union after failed traction. The achieved favorable outcomes, documented through meticulous case reporting and a literature review, provide critical clinical insights for managing such complex pathologies.

## 2. Case Presentation

### 2.1. History and Physical Examination

The patient details were de-identified. A male patient in his 50s presented with neck pain and restricted mobility for 25 days following a traumatic fall incident. The injury occurred when the patient, under alcohol intoxication, experienced a descent down stairs, resulting in head collision with a wall, resulting in immediate severe head and neck pain. No extremity paralysis or loss of consciousness was reported. Initial assessment at a local hospital diagnosed “fractures involving both the anterior and posterior arches of the atlas vertebra.” The patient declined surgical intervention and opted for conservative management utilizing cervical brace immobilization. Due to persistent symptoms, the patient was admitted for further therapeutic intervention. The patient’s medical history was unremarkable except for a 30-year history of smoking and alcohol consumption.

Specialized physical examination revealed significantly restricted neck and right shoulder movement, mild leftward head tilt, no obvious superficial or deep sensory deficits in the limbs, mild weakness (grade 4) of the intrinsic hand muscles, and preserved muscle strength in other limb muscle groups. Bilateral radial periosteal reflexes, biceps reflexes, and triceps reflexes were hyperactive. Hoffmann’s sign was negative bilaterally, and Babinski’s sign was positive on the left and negative on the right. Preoperative laboratory and imaging studies confirmed the diagnosis of “Gehweiler type IIIb atlas fracture [9]; right atlantoaxial lateral mass joint dislocation; lacunar cerebral infarction; carotid atherosclerosis; lower extremity atherosclerosis; hepatic steatosis; left hepatic lobe cyst; and right rotator cuff injury.” The VAS score for neck pain was 4, and the NDI was 64%.

### 2.2. Imaging Findings

**Figure 1:**
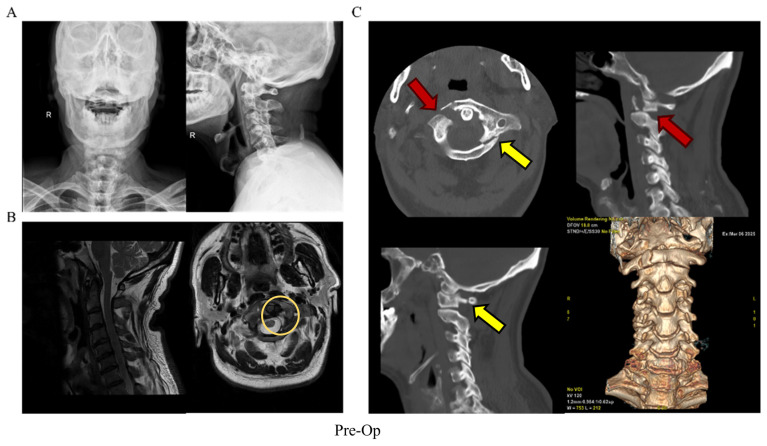
Preoperative imaging findings: (**A**) Anteroposterior and lateral radiographs of the cervical spine. (**B**) Cervical spine MRI demonstrating complete transverse atlantal ligament (TAL) rupture (yellow circle), mild cervical intervertebral disk protrusion, and no spinal cord compression. (**C**) Cervical spine CT showing a displaced fracture at the transitional region between the right anterior arch and lateral mass of the atlas combined with atlantoaxial dislocation (red arrow), as well as a fracture of the left posterior arch (yellow arrow).

Cervical X-ray (open-mouth projection) demonstrated asymmetric atlantodental intervals along with degenerative alterations in the cervical spine. CT imaging revealed a displaced fracture at the transitional zone between the right anterior arch and lateral mass of the atlas with concomitant atlantoaxial dislocation, along with a left posterior arch fracture. MRI showed complete rupture of the transverse atlantal ligament (TAL), mild cervical disk protrusion, and no evidence of spinal cord compression (Figure 1).

### 2.3. Hospital Management

**Figure 2:**
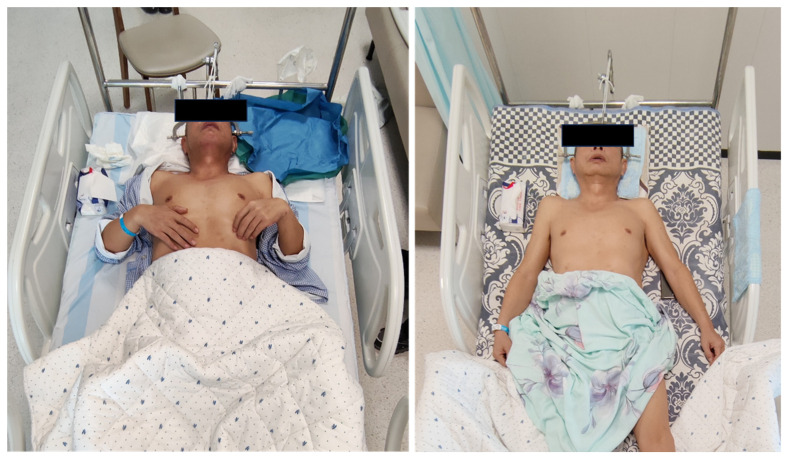
Clinical photographs of the patient before traction (**left**) and after 1 week of 8 kg skull traction (**right**).

**Figure 3:**
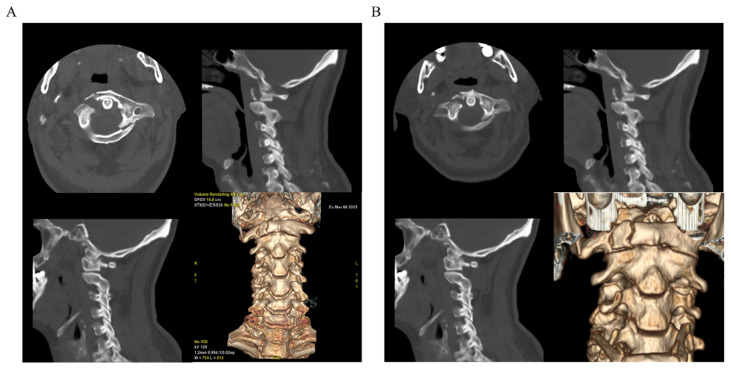
CT imaging of pre-traction (**A**) and after 8 kg traction (**B**).

Following admission, the patient received symptomatic management including analgesics and neurotrophic agents. Preoperative oral preparation was performed using chlorhexidine mouthwash bid, and oral metronidazole 0.2 g tid. Skull traction was initiated for fracture reduction, commencing at 4 kg and subsequently incremented to 8 kg. Post-traction imaging demonstrated partial correction of the head tilt deformity (Figure 2). However, cervical CT under traction revealed persistent atlantoaxial dislocation, confirming an irreducible status (Figure 3). A 3D-printed atlantoaxial complex was generated for preoperative planning.

### 2.4. Operative Technique

**Figure 4:**
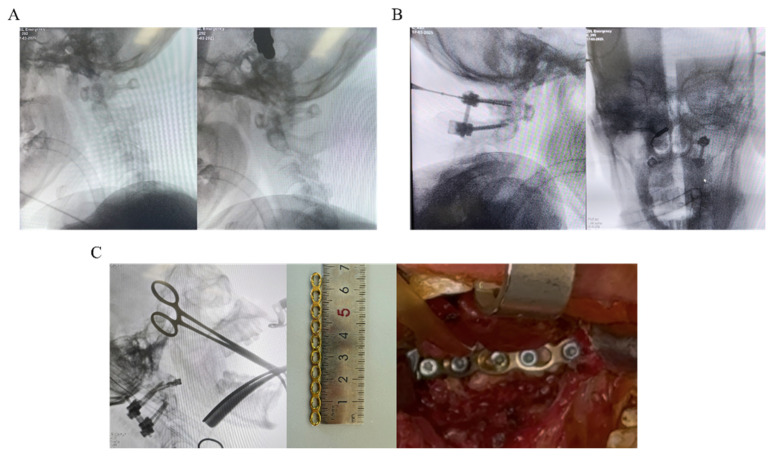
Intraoperative imaging examinations: (**A**) Lateral cervical radiographs acquired before and after skull traction. (**B**) Lateral cervical radiograph during posterior internal fixation. (**C**) Anterior approach intraoperative imaging.

The surgical procedure incorporated posterior atlantoaxial open reduction in conjunction with internal fixation (ORIF) with anterior transoral atlantoaxial ORIF. After induction of general endotracheal anesthesia, the patient was positioned in a prone orientation with cervical flexion stabilized by a Mayfield head clamp, and continuous 8 kg cranial traction was applied to achieve partial fracture reduction (Figure 4A).

### 2.5. Posterior Approach

A midline incision from C1 to C3 exposed the C1 posterior arch, C2 spinous process, laminae, and bilateral lateral masses. Pedicle screw entry trajectories were prepared using a drill guide, followed by pilot hole tapping and K-wire placement under fluoroscopic visualization. Four pedicle screws (C1 and C2) were inserted bilaterally with satisfactory positioning confirmed intraoperatively (Figure 4B). Hyperplastic fibrous tissue indicative of delayed union was identified at the left C1 posterior arch fracture site. Fibrous adhesions were thoroughly debrided, fracture margins were contoured, and displaced bone fragments were repositioned to reduce lateral mass dislocation. After achieving adequate hemostasis, the lateral mass joint space on the dislocated side was explored to confirm reduction. Pre-contoured titanium rods were secured to the screw heads using locking caps. The left C1 posterior arch fracture site was decorticated with a high-speed burr. Autologous cancellous bone harvested from the right posterior iliac crest, combined with synthetic bone graft, was implanted across the prepared C1-C2 fusion bed. A closed suction drain was placed prior to layered closure.

### 2.6. Anterior Transoral Approach

After repositioning the patient supine with neck extension, bilateral soft palate retraction provided exposure of the posterior pharyngeal wall. Thorough irrigation with povidone-iodine solution preceded a midline longitudinal incision (3.5 cm) through the pharyngeal mucosa. The anterior longitudinal ligament and longus colli muscles were divided to expose the C1 anterior arch and upper C2 vertebra. Intraoperative exploration confirmed the anterior arch fracture, accompanied by two fracture lines, and the detached bone fragments had formed fibrous adhesions. Given that fibrotic tissue severely impedes fracture healing and isolated internal fixation fails to facilitate bony reconstruction at the junction of the anterior atlas arch and lateral mass articulation, all the fibrous adhesions at the fracture site were carefully removed. Fibrotic tissue was meticulously debrided using curettes. After fracture site decortication, cancellous autograft and allograft were packed into the defect. A pre-bent locking plate was anchored to the anterior arch using self-tapping locking screws under fluoroscopic guidance (Figure 4C). Mucosal closure was performed in layers. Total operative time: 5 h; intraoperative blood loss: 300 mL.

Posterior C1/C2 pedicle screw system: Manufactured by WEGO Medical. All screws had a diameter of 3.5 mm. C1 pedicle screws were 22 mm in length bilaterally; C2 pedicle screws measured 24 mm (right) and 26 mm (left).

Anterior transoral locking plate system: Manufactured by RUIHE Medical. A 2.0 mm ten-hole locking plate was applied, with five locking screws of varying lengths: one 10 mm screw, two 12 mm screws, one 16 mm screw, and one 18 mm screw. Under fluoroscopic guidance, screws were placed perpendicular to the anterior arch of the atlas and the screws inserted on left and right sides were obliquely directed toward the bilateral C1 lateral masses.

Intraoperative neuromonitoring: No SSEP or MEP monitoring was implemented throughout the operation.

### 2.7. Postoperative Care

**Figure 5:**
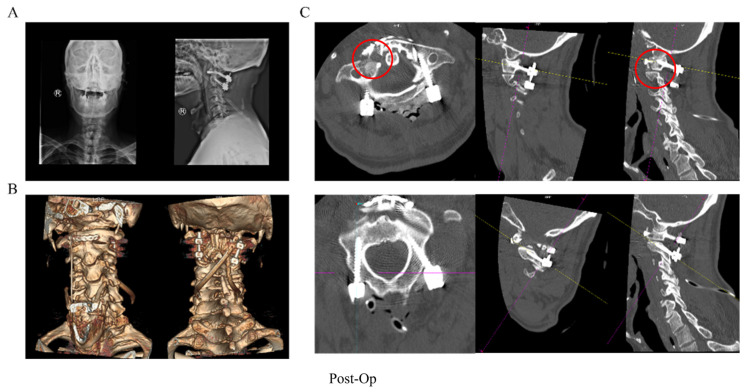
Postoperative imaging assessments: (**A**) Anteroposterior and lateral radiographs of the cervical spine. (**B**) Postoperative 3D reconstruction CT image. (**C**) Postoperative cervical CT; fracture sites and dislocated C1 lateral mass joints were reduced and fixed (red circle).

The patient was transferred to the ICU with an endotracheal tube following surgical intervention. Upon restoration of consciousness and demonstration of adequate spontaneous respiratory function, the patient was discharged from the ICU. Hospital-acquired pneumonia developed during the convalescent period and was successfully managed with antibiotic therapy. Follow-up imaging confirmed satisfactory implant positioning (Figure 5). The neck pain VAS score decreased from 4 preoperatively to 2, and the patient regained independent ambulation capabilities, with satisfactory wound healing and gait improvement observed prior to discharge.

### 2.8. Postoperative Follow-Up

**Figure 6:**
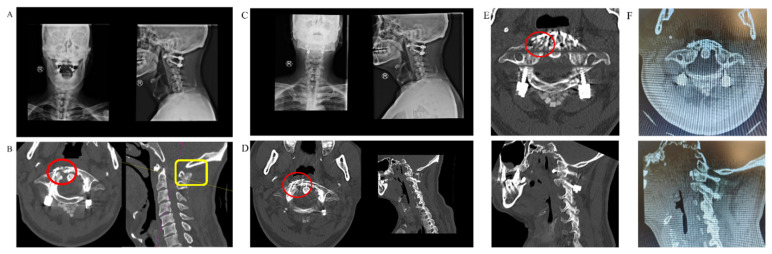
Follow-up imaging: (**A**) Anteroposterior and lateral cervical radiographs at 1 month postoperation. (**B**) Atlas CT at one month postoperation. (**C**) Cervical spine anteroposterior and lateral radiographs at two months postoperation. (**D**) Atlas CT at two months postoperation. (**E**) Atlas CT at three months postoperation. (**F**) Atlas CT at nine months postoperation. Red circle: fracture of the anterior atlas arch; yellow square: posterior atlantal bone graft region.

**Figure 7:**
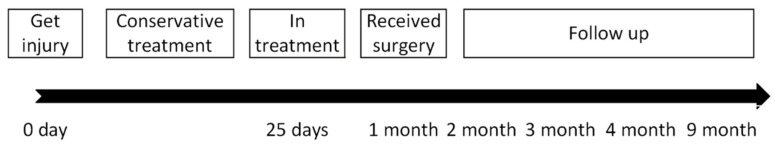
The time line of patient’s treatment.

The patient wore a Philadelphia cervical orthosis continuously for 3 months after surgery; during the initial three-month follow-up period, the patient’s oral and posterior cervical incisions demonstrated satisfactory healing. At the 9-month follow-up, the patient’s VAS score decreased to 0, and the NDI decreased to 16%. Cervical open-mouth radiographs demonstrated improved symmetry of the atlantodental intervals compared to preoperative measurements. Cervical CT revealed that the fracture line at the junction of the anterior arch and the lateral mass had almost completely healed. Osseous fusion was observed at the dorsolateral graft site of the posterior arch, and no signs of implant loosening were detected during the nine-month follow-up period (Figure 6). At the 3-month visit, the patient reported the ability to complete all routine daily activities independently. At the 9-month follow-up, he nearly returned to his pre-injury functional level, with only mild limitation in cervical rotation (bilateral cervical rotation: 40°) that did not interfere with daily living (Figure 7, Table 1).

### 2.9. Postoperative Rehabilitation Strategy

The patient was instructed to wear a Philadelphia cervical orthosis for the first 3 postoperative months to facilitate solid bony fusion. After 3 months, once bony union became relatively stable, the orthosis could be removed, and structured cervical muscle rehabilitation was initiated, including gentle cervical stretching and low-resistance strengthening exercises. Training intensity was increased gradually, with instructions to avoid any significant discomfort after each session. Functional training was discontinued at 6 months postoperation, allowing the patient to resume full daily activities.

## 3. Discussion

Electronic literature retrieval was performed across the biomedical databases PubMed/MEDLINE and Web of Science from database inception to May 2026. Boolean search strings combining MeSH terms and free-text keywords were applied for retrieval. The full English Boolean search formula was as follows: (atlas OR C1 OR Jefferson) AND fracture AND (transoral OR anterior cervical OR posterior atlantoaxial fixation).

Reference lists of all included articles were manually screened to retrieve additional relevant studies. Two independent spine surgeons conducted two-stage literature screening (title and abstract screening first, followed by full-text evaluation). Any disagreements during screening were resolved via discussion or consultation with a senior attending surgeon. The whole screening process complied with the PRISMA statement.

Inclusion Criteria: (1) Human adults (≥18 years) with traumatic atlas fractures. (2) Studies describing transoral anterior, posterior, or combined anteroposterior surgical fixation. (3) Original clinical studies: case reports, case series, cohort trials with complete clinical/radiological follow-up data.

Exclusion Criteria: (1) Animal, cadaveric, finite element biomechanical studies. (2) Non-traumatic, pediatric, pure conservative treatment or irrelevant cervical injuries. (3) Conference abstracts, letters, duplicate publications, papers missing core follow-up data.

Atlas burst fractures were first described by Jefferson in 1920 [10]. Due to the atlas’s unique force [2], fracture fragments typically displace outward, rarely encroaching on neural structures. Current management strategies for atlas fractures remain controversial [11]. Conservative external immobilization is often used for stable fractures with intact TAL [3,8], but 20–80% of patients have residual neck pain after long-term immobilization [3]. A long-term follow-up of Jefferson fracture patients revealed incomplete restoration of pre-injury health status compared to population norms [12]. Furthermore, delayed reduction in unstable injuries with concomitant ligamentous disruption may lead to irreversible atlantoaxial incongruity, joint stiffness, and exacerbated pain [13], challenging the rationale for conservative prioritization. Spence proposed lateral mass displacement > 6.9 mm on radiographs as a criterion for TAL incompetence and fracture instability [14], though Dickman et al. contested this, noting concordance in only 39% of cases [15]. Recent systematic reviews highlight persistent ambiguity in defining upper cervical instability, emphasizing the necessity of advanced imaging for conclusive assessment [16]. In our case, MRI confirmed TAL avulsion without bony involvement (Dickman Type I injury) [15]. As the TAL is the primary stabilizer against anterior C1-C2 translation [17], its midsubstance failure (Type I) precludes effective external stabilization. The concomitant anterior arch-lateral mass junction fracture, posterior arch fracture–dislocation, and Gehweiler IIIb classification collectively indicated mechanical instability.

For unstable atlas fractures, posterior C1-C2 fusion techniques (e.g., Goel, Magerl, Harms, Wright) remain mainstream [1,11,18]. Emerging posterior approaches, including C1 laminar hooks with C2 screws [19] and robot-assisted percutaneous lag screw fixation [20], have shown efficacy. However, C1-C2 fusion inevitably compromises cervical rotation. Recent trends favor motion-preserving C1 standalone fixation [18,21,22,23,24], with a prospective multicenter study demonstrating superior long-term pain relief and functional outcomes compared to fusion [25]. Nevertheless, posterior-alone fixation inadequately stabilizes anterior arch fractures [4]. Innovative posterior unilateral compression screw techniques for anterior arch fixation have been explored [26] yet have not achieved widespread clinical adoption. Anterior transoral approaches enable direct anterior column reconstruction, with evolving fixation strategies ranging from rod-screw constructs to plate systems and unilateral lag screws [4,6,27,28,29,30]. This minimally invasive route minimizes myofascial disruption, and meticulous perioperative protocols effectively mitigate infection risks. However, isolated C1 fixation carries inherent redislocation risks [18], and evidence remains limited by small cohorts and the paucity of comparative studies [31], furthermore, there is a notable absence of long-term follow-up data regarding fixation intensity. Therefore, in elderly patients with complex atlas fractures and irreducible lateral mass dislocation, the efficacy of C1 fusion alone has not yet been fully established.

No consensus exists for optimal Gehweiler IIIa/IIIb fracture management [18]. Prior research has recommended atlantoaxial fusion [9]. Our patient’s longitudinal anterior arch-lateral mass split with posterior arch fracture–dislocation (Gehweiler IIIb) exhibited translational instability, for which some advocate for C1-C2 fusion as a suitable choice, particularly in elderly patients [32]. Bhandutia et al. posit that while standalone C1 fixation offers motion-preserving potential, its benefits are maximized in younger patients with isolated injuries or low-complexity polytrauma, particularly when considering potential revision surgeries. For elderly patients, posterior C1–C2 fusion remains a better option [31]. According to Denton H’s research, C1–2 fusion remains the optimal solution for unstable type IIIb fractures, as the rupture of the transverse atlanto-occipital ligament leads to atlantal instability [18]. Radiomorphologically, this patient’s fracture pattern bears similarity to the atlas fracture subtype described by Bransford et al. [33], which is frequently associated with delayed cock-robin deformity and intractable pain. Eight-kilogram cranial skull traction failed to reduce the lateral mass joint, a finding explained by intraoperative confirmation of fibrous scar ingrowth across the fracture margins. Given this chronically irreducible atlas fracture refractory to conservative management, we adopted an aggressive combined surgical protocol consisting of posterior C1–2 arthrodesis with iliac autograft plus anterior transoral locking plate–screw fixation, although this procedure results in partial loss of cervical mobility. Unlike previously described transoral lateral mass screw techniques [4,6,8], our method applies orthopedic trauma principles for multiplanar rigid spinal fixation. Compared with custom implants, orthopedic trauma osteosynthesis plates feature easy accessibility and lower cost. Multiple compression screws anchor both fracture fragments and native bone to restore anatomical alignment. Concurrent posterior C1–2 fusion was added to reduce stress concentration on the anterior implant construct. Early follow-up revealed satisfactory biomechanical and clinical results. While C1–2 fixation and fusion inevitably reduce cervical range of motion, rigid stabilization and bony fusion took priority over preserving mobility in this patient with a complex, long-standing atlantoaxial injury. Notably, the combined anterior–posterior approach taken for this rare case is associated with greater operative trauma, transoral infection, and implant exposure from pharyngeal dehiscence; thorough preoperative evaluation is required prior to surgery.

The patient developed hospital-acquired pneumonia following surgical intervention as a consequence of a documented smoking history and endotracheal intubation. Infection represents a significant clinical challenge in transoral anterior cervical spine procedures, attributable to suboptimal wound healing characteristics of the posterior pharyngeal wall and the presence of oral bacterial colonization. Such infectious complications may result in internal fixation failure, thereby necessitating comprehensive perioperative oral hygiene protocols. Patients utilize chlorhexidine and Anerdian mouth rinses postoperatively alternately every hour, with intraoperative Anerdian irrigation administered at 15 min intervals. Although prior clinical reports indicate favorable prognostic outcomes, meticulous monitoring for potential complications associated with prolonged intubation remains imperative. Tracheal extubation should be promptly implemented upon restoration of alertness, resolution of laryngeal edema, and establishment of spontaneous breathing patterns, followed by subsequent transfer to a general ward setting.

## 4. Conclusions

The uniqueness of this case report lies in the innovative application of orthopedic trauma principles through an anterior transoral locking plate–screw construct, which provides robust rigid fixation for chronic atlas fractures, solving the problem of the anterior arch requiring a specially customized internal fixation device. When combined with posterior C1–2 arthrodesis, this hybrid strategy represents an optional surgical solution for managing irreducible chronic atlas fracture–dislocations, achieving both anatomical stabilization and biomechanical durability during the short-term follow-up after surgery, which could be considered in highly specific, refractory cases managed in specialized centers, and it also requires extensive clinical verification.

## Figures and Tables

**Table 1:** The time line of VAS and NDI assessments.

Timing Point	VAS	NDI
Preoperation	4	64%
Hospital discharge	3	52%
1-month follow-up	2	36%
3-month follow-up	2	24%
9-month follow-up	0	16%

## Data Availability

All clinical follow-up data and imaging materials related to the patient have been fully presented within the main text of the article.
